# Supplementary material for: Eukaryotic stress–induced mutagenesis is limited by a local control of translesion synthesis
Source: Nucleic Acids Res. 2022 Feb 1;50(4):2074–80. doi: 10.1093/nar/gkac044 (PMC8887424; doi:10.1093/nar/gkac044)
Supplement: gkac044_Supplemental_File [file gkac044_supplemental_file.pdf]

Eukaryotic stress induced mutagenesis is limited by a local control of Translesion Synthesis

---

Katarzyna H. Masłowska[1], Florencia Villafañez[1], Luisa Laureti[1], Shigenori Iwai[2], Vincent Pagès[1*]

[1]Cancer Research Center of Marseille: Team DNA Damage and Genome Instability | CNRS, Aix Marseille Univ, INSERM, Institut Paoli-Calmettes, Marseille, France
[2]Graduate School of Engineering Science, Osaka University, Osaka, Japan
[*]vincent.pages@cnrs.fr

Supplementary data

Supplementary Figure 1:

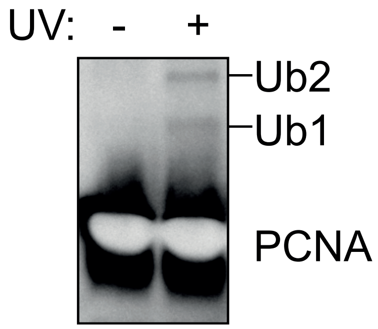

Supplementary Figure 1: Western-blot analysis for the UV irradiation condition revealing a significant increase in PCNA ubiquitination in the treated condition.

Supplementary Figure 2:

HPLC control of the (6-4)TT photoproduct oligonucleotide

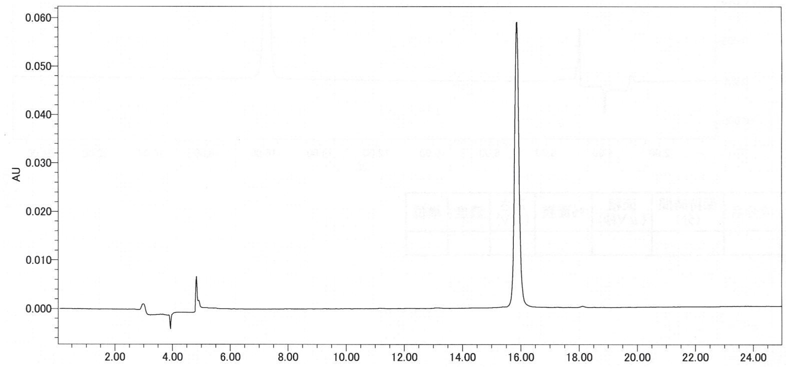

Supplementary Figure 2: Chromatogram showing the purity of the (6-4) TT photoproduct oligo following 2 chromatography columns (as describe in the method section).
